# A Comprehensive Review of Non-Conventional Yeasts: Innovation in Craft Beer Production

**DOI:** 10.3390/foods15020253

**Published:** 2026-01-10

**Authors:** Laura Canonico, Francesca Comitini, Alice Agarbati, Maurizio Ciani

**Affiliations:** Department of Life and Environmental Sciences, Polytechnic University of Marche, Via Brecce Bianche, 60131 Ancona, Italy; f.comitini@univpm.it (F.C.); a.agarbati@univpm.it (A.A.)

**Keywords:** non-conventional yeasts, management, functional beer, aroma profile

## Abstract

The craft beer market is continually expanding, driven by the consumers’ demand for product diversification, which leads to innovation in the brewing industry. While traditional brewing focuses on consistency and high-volume efficiency using standard yeasts, craft brewing prioritizes small-batch experimentation and flavor complexity. Traditionally, *Saccharomyces cerevisiae* (Ale beer) and *Saccharomyces pastorianus* (Lager beer) yeast are used in brewing. The craft brewing revolution introduced the use of non-conventional yeast. These yeasts possess distinct technological characteristics compared to commercial starters, such as a richer enzyme profile. This biological diversity produces beers with novel, complex aroma profiles, and opens exciting avenues for flavor creation. Recently, non-alcoholic beer and low-alcoholic beer (NABLAB), and functional beer have become the new horizons for the application of non-conventional yeasts. In recent years, the brewing potential of these alternative yeasts has been extensively explored. However, some aspects relating to the interactions between yeast and raw materials precursors involved in the aroma of the final beer, and the management of yeasts in fermentation, remain unexplored. This review systematically outlines the various innovative ways in which non-conventional yeasts are applied in brewing, including healthier beer. Here, we explore how these yeasts can foster innovation in the beer sector and provide the possibility for sustainable development in contemporary brewing.

## 1. Introduction

The craft brewing phenomenon has seen significant growth since the early 2000s, piquing consumer interest and transforming the industry. Following an extended period dominated by a few global multinationals, craft breweries emerged, introducing innovative products and experimenting with diverse ingredients and brewing processes [1]. Craft beer stands out as a dynamic and creative sector within the food and beverage industry, focusing on quality, aroma, health, sustainability, regionality, and customized brewing technologies [2].

**Figure 1:**
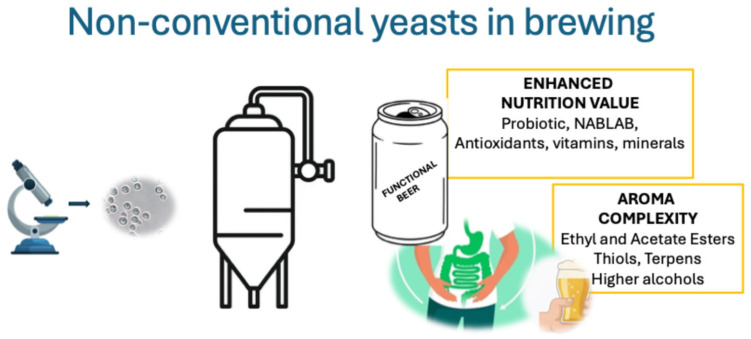
The main application of non-conventional yeasts in craft beer.

The creation of high-quality beer relies on brewing yeasts that yield positive fermentation as well as desirable aroma and flavor profiles [3]. The importance of *Saccharomyces* and non-*Saccharomyces* yeasts extends beyond the production of ethanol and carbon dioxide; they also generate various intermediate compounds and by-products that contribute to the taste and aroma of the beer [3,4]. In the 19th century, the industrial brewing sector’s demand for more uniform products led to the development of specific strains of brewer’s yeast, such as *Saccharomyces cerevisiae* for Ale beer and *Saccharomyces pastorianus* for Lager beer. Only a few beer styles, such as Belgian lambic and American Coolship ales, utilize spontaneous fermentation, incorporating native microorganisms, including non-conventional yeast species and bacteria. Research into indigenous non-conventional yeasts represents a promising strategy for craft beers (Figure 1) with unique characteristics.

These yeasts can offer a variety of aromatic compounds and functional benefits, facilitating the production of innovative beer styles [5]. Non-conventional yeasts can diversify products, creating new beer styles, including flavored varieties, as well as specialty beers [6,7]. In addition to the classic styles, so-called specialty beers are appearing in the market, including products that do not fall into conventional styles, such as low-calorie, low-alcohol, and/or non-alcoholic (NABLAB) and gluten-free beers [8].

This review systematically explores the use of non-conventional yeasts in craft beer production. These yeasts contribute significantly to beer fermentation by enhancing flavor profiles through pure and mixed fermentation with *S. cerevisiae* and by producing organic acids in sour beers. Furthermore, non-conventional yeasts can modulate hop aromas, enhancing fruity perceptions through the release of thiols or terpenes via enzymatic actions and reducing off flavors like diacetyl or acetaldehyde. Finally, we will discuss the potential uses and applications of non-conventional yeasts in producing NABLAB and functional beers and the potential limitations due to the poor knowledge, particularly on safety aspects.

## 2. Non-Conventional Yeast in Brewing

Non-conventional yeast strains with brewing potential can originate from various sources, including official microorganism collections and natural environments like fermentative ecosystems, sourdough fermentation, and kombucha [8,9,10] and even isolated from contaminating yeasts in breweries that possess valuable brewing capabilities [11]. The current market demands a broader diversity of beers produced efficiently and sustainably, necessitating a new approach to yeast selection in fermentation. The traditional industrial brewing works with high-volume efficiency using standard yeasts. The final beer must be a consistent product over time to build consumer loyalty. Differently, craft brewing prioritizes small-batch experimentation and specific and special beers with flavor complexity. Traditionally, *S. cerevisiae* (Ale beer) and *S. pastorianus* (Lager beer) yeast are used in brewing. The craft brewing revolution managed the use of non-conventional yeast. Relying solely on commercial starter strains is no longer adequate for achieving these goals, particularly for low-alcohol beer production. As consumer interest shifts towards new flavor profiles, where alcohol content is less critical, the industry must embrace a wider range of yeast species. This shift reflects a growing enthusiasm for beers beyond traditional styles, which calls for redefining the concept of “brewer’s yeast” and expanding the variety of strains used in breweries [12].

### 2.1. Management of Non-Conventional Yeasts in Brewing

Non-conventional yeasts can be employed in spontaneous fermentation, mixed fermentation, co-fermentation, or as pure cultures in beer production (Table 1). Their different carbohydrate metabolism allows their application in conventional, low-alcohol, and non-alcoholic beers [6]. By positively influencing the sensory attributes of beer, these yeasts enhance aroma and flavor, serving as a natural tool for creating new styles that align with consumer demand for health beverages [7]. The fermentative performance of yeast during brewing is influenced by various factors, including wort ion concentration, stress tolerance, wort density at inoculation, dissolved oxygen levels, and fermenter geometry. Understanding these factors is essential for selecting an appropriate yeast strain for beer production. To ensure quality, the chosen yeast must efficiently metabolize nutrients from the wort, withstand environmental conditions such as osmotic pressure, pH, temperature, and ethanol concentration, and impart the desired flavors to the beer (Table 1). Due to the extensive biodiversity of yeast strains available, particularly those derived from diverse habitats such as wild and fermented foods and beverages [13], research on managing non-conventional yeasts is still in its early stages. However, several studies have begun to evaluate the use of various non-conventional yeast species in pure, mixed, sequential, or spontaneous fermentation cultures.

### 2.2. Spontaneous Fermentation

Spontaneously fermented beer is characterized by the wort without actively introducing any fermentative microbes by the brewer [34]. The initial fermentation occurs spontaneously, driven by wild yeasts and environmental bacteria. Following this primary fermentation, the brewer may introduce a specific culture of one or more microorganisms for secondary fermentation. Traditional examples of spontaneously fermented beers include Belgian lambics and sour lambics, which were originally crafted in the Senne Valley, particularly around Brussels, between October and March [35,36].

In the lambic brewing process, after mashing, the wort is transferred to open, shallow metal vessels known as coolships, where it is left to cool overnight. During this cooling period, microorganisms from the atmosphere inoculate the wort, initiating fermentation [5]. These coolships are typically stored in wooden-roofed rooms to allow the water vapor from the hot wort to condense and drip back into the vessel. Once cooled, the wort is transferred to wooden barrels, where fermentation continues at room or cellar temperatures, ranging from 15 to 25 °C. The microbiota present in these barrels also contributes to the fermentation process [5,37].

Maturation occurs in the same wooden barrels for a period ranging from 1 to 3 years before bottling. The final product is a sour, cloudy beer with an alcohol content of approximately 5%, low carbonation, and a light head [38]. Aging enhances certain aromatic characteristics, and achieving a harmonious balance among these flavors is crucial for consumer enjoyment. Spontaneous fermentation involves a complex and dynamic microbiota, with variations arising from different geographical regions, brewing techniques, and recipes [5,39,40,41].

#### Phases of Spontaneous Fermentation

Spontaneous fermentation can be divided into four distinct phases, each marked by a unique microbial consortium.

1. Initial Phase (1–4 weeks): This phase is dominated by Enterobacteriaceae [42,43], along with oxidative yeasts from genera such as *Candida*, *Cryptococcus*, *Debaryomyces*, *Hansenula*, *Pichia*, and *Rhodotorula* [44].

2. Main Fermentation Phase: Here, yeasts from the *Saccharomyces* genus, including *S. cerevisiae*, *S. bayanus*, *S. kudriavzevii*, and *S. pastorianus*, become predominant [40]. Other genera, such as *Hanseniaspora* and *Kazachstania*, are also present [44]. During this phase, these yeasts produce secondary metabolites—including esters, higher alcohols, and organic acids that significantly influence the organoleptic profile of beers [17,44].

3. Acidification Phase: In this phase, the fermentation is dominated by Lactobacilli and Pediococci, alongside lactic acid bacteria (LAB) [37,44,45]. Here, *Saccharomyces* yeasts are gradually replaced by *Brettanomyces* spp., which also play a crucial role during the maturation phase [46].

4. Maturation Phase: This final stage can last from several months to years and is primarily characterized by the activity of *Brettanomyces* yeasts, which exhibit high ethanol tolerance and the ability to ferment complex carbohydrates that are typically unfermented by *Saccharomyces* spp. [36,40,45]. Recent research indicates that multiple strains or subspecies of *Brettanomyces* are involved in wild beer production [41]. These yeasts impart the characteristic “Brett character” to the beer, often described as a mousy aroma with notes ranging from stable and horsey to leathery, medicinal, smoky, and clove-like. Additionally, *Brettanomyces* yeasts can produce fruity and floral aroma compounds through the bioconversion of hop compounds, wort carbohydrates, and exopolysaccharides from Pediococci [40,46].

Recent advancements in understanding the various microorganisms involved in spontaneous fermentation have led to the development of innovative techniques for sour beer production. These techniques enable better process control and reduce production time. One such approach involves the controlled use of non-conventional yeasts in mixed fermentation with *S. cerevisiae* strains, resulting in beers that possess unique sensory characteristics alongside their sourness. For instance, a Lambic beer was produced using a combination of a *Dekkera bruxellensis* (synonymous of *Brettanomyces bruxellensis*) strain, the commercial starter yeast *S. cerevisiae* S-04, and the LAB *Lactobacillus brevis*, all co-fermented together [47]. This co-fermented beer demonstrated a significant enhancement in aromatic characteristics and antioxidant capacity compared to sour beers inoculated solely with *S. cerevisiae*. After 12 months, the beer exhibited a pronounced “Brett” aroma profile and increased antioxidant properties [47].

### 2.3. Pure Culture

Non-conventional yeasts are increasingly applied in brewing to modulate fermentation performance, aroma formation, and sensory properties, and many species can be used in pure culture to produce beers with low or no alcohol (Table 1). Their use has been investigated primarily to enhance flavor complexity and overall sensorial quality. Among them, *Wickerhamomyces anomalus* (Table 1) and *Torulaspora delbrueckii* (Table 1) are considered particularly promising for beer production, as they generate distinctive ester and higher alcohol profiles while often limiting ethanol formation. *W. anomalus* ferments maltose with strain dependent variability and is an efficient producer of ethyl propanoate, β-phenyl ethanol, 2-phenyl ethyl acetate, and especially ethyl acetate, which can contribute pleasant fruity notes at moderate levels but solvent like aromas when excessive [25,26,27]. *T. delbrueckii* has also been widely studied in brewing; it can show variable malt fermenting ability, but typically yields beers rich in amyl alcohols and esters associated with rose, bubblegum, banana, and citrus notes, combined with low attenuation (37%) and reduced ethanol content (2.66% *v*/*v*), making it attractive for low alcohol styles [48]. Several other non-conventional yeasts have shown valuable brewing traits. *Pichia kluyveri* can produce very high levels of 2-phenyl ethyl acetate, isoamyl acetate, ethyl acetate, and linalool [49] whereas *Pichia anomala* and *Pichia kudriavzevii* tend to generate high ethyl acetate but comparatively low isoamyl acetate, creating different fruity profiles The use of *P. kluyveri*, as a starter culture to produce beer with highly interesting aroma characteristics, in need of optimizing the process conditions and/or selecting the most suitable *S. cerevisiae* strains for the second inoculation. *Zygoascus meyerae*, *P. anomala* can significantly exceed sensory thresholds for key volatiles such as 4-vinylguaiacol, β-phenyl ethanol, and geraniol, resulting in beers with pronounced clovelike-, floral, and fruity characteristics [50]. A study on *P. kluyveri* and *Hanseniaspora vineae* showed significant increases in free 3-mercaptohexan-1-ol (3MH), enhancing passion fruit notes [28]. Moreover, some researchers found that the high production of acetate esters by these same yeasts can create a “masking effect,” where the heavy floral/banana scents overwhelm the delicate tropical thiols A strain of *Candida glabrata*, which exhibited high β-glucosidase activity, showed a 50-fold higher geraniol content, resulting in the production of a final beer with unique floral and fruity characteristics [51]. *Lachancea thermotolerans* (Table 1) contributes to lactic acid during primary fermentation, imparting the sourness and mouthfeel typical of sour beers [20]. However, higher concentrations of lactic acid can lead to a perceived loss of “mouthfeel” and can inhibit the expression of certain hop-derived terpene aromas, making the beer taste “thin” or “sharp” rather than complex [52].

*Brettanomyces/Dekkera* species (particularly *B. bruxellensis*) are notable for producing complex mixtures of esters and phenolic compounds that can add spicy, fruity, or “funky” notes valued in certain specialty beers when present at controlled levels. It is widely demonstrated that small amounts of 4-EG and 4-EP are seen as essential for the “terroir” and complexity of wild ales and Lambics. In contrast, in many craft styles (like NEIPAs), even trace amounts of these compounds are classified as spoilage. Furthermore, the vinyl phenol reductase (VPR) enzyme activity varies so wildly between strains that results are often unpredictable [53].

Their β-glucosidase activity enables hydrolysis of glycosidic precursors in hops and wood, releasing monoterpenes such as linalool and methyl salicylate that intensify citrus, floral, minty, or spicy hop aromas [54]. Selected strains are therefore attractive both for pure fermentations and for co-culture with *S. cerevisiae*. Pure fermentation of the following non-conventional yeast species, *H. vineae*, *Hanseniaspora valbyensis*, *Metschnikowia pulcherrima*, *Hanseniaspora guilliermondii*, *Zygosaccharomyces bailii*, *T. delbrueckii*, *W. anomalus* was carried out [55]. Among these, *W. anomalus* was selected in pure culture for brewing low alcohol content beer (1.25% (*v*/*v*) for its fruity and phenolic flavors and the absence of wort flavors. Other non-conventional yeast species, unable to ferment maltose, may be used in sequential or mixed fermentations to contribute to flavor profiles. *Saccharomyces boulardii* investigated in pure culture for several applications produced probiotic non-alcoholic beer [56]. In a study of Breno Pereira de Paula et al. [57], it was reported that *S. boulardii* has the potential to produce probiotic wheat beers, without any significant reduction in the population during beer storage, tolerating typical wort sugars and bitterness and providing potential health benefits linked to probiotic intake. Thus, the experimental results mentioned above demonstrate that *S. boulardii* can brew probiotic non-alcoholic beer [56]. Overall, these findings support the use of non-conventional yeasts in pure, sequential, or mixed fermentations as valuable tools for creating beers with reduced alcohol content, enhanced complexity, and potential functional properties.

### 2.4. Sequential Fermentation

Sequential and mixed fermentations with non-conventional yeasts and *S. cerevisiae* are widely used to diversify beer flavor, enhance aroma complexity, and, in some cases, improve functional properties such as antioxidant activity and probiotic potential [57]. These approaches allow brewers to combine the metabolic traits of different yeasts to obtain beers with distinctive sensory profiles and added health -related value [17,58,59,60]. Non-conventional yeasts are often co-inoculated or used sequentially with *S. cerevisiae* to ensure reliable fermentation while enriching the volatile profile with esters, higher alcohols, phenols, and other aroma-active metabolites. The final aroma of each beer depends strongly on the chosen strain combination and on process parameters such as inoculation order, inoculum ratio, temperature, and fermentation time.

*T. delbrueckii* is regarded as one of the most promising non-conventional yeast species for brewing, particularly in mixed or sequential fermentations [61]. When used first and followed by *S. cerevisiae*, it can increase compounds like 4—vinyl guaiacol and modify phenolic and ester balances, yielding beers with enhanced spicy or clove -like notes and altered fatty-acid derived flavors [28]. Co-fermentation at different *S. cerevisiae*/*T. delbrueckii* ratios can shift production of esters such as ethyl octanoate and modulate levels of acetaldehyde and higher alcohols, providing a practical lever for fine-tuning aroma [62]. Kayadelen et al. [63] demonstrated that the ester content of beer produced by mixed fermentation of *T. delbrueckii* with *S. cerevisiae* is higher than that produced by *S. cerevisiae* alone.

*P. kluyverii* and *Pichia kudriavzevii* have attracted attention to their strong impact on fruity esters in mixed or sequential fermentations. *P. kluyveri* can markedly increase isoamyl acetate and related compounds, intensifying banana-like notes that are desirable in wheat beers, while co-fermentation with *S. cerevisiae* helps complete attenuation [28]. In a recent work this species showed in both co-culture and sequential fermentation residual sugar levels comparable to those exhibited by *S. cerevisiae* monoculture fermentation [49]. *P. kudriavzevii*, when combined with *S. cerevisiae*, tends to produce high levels of ethyl acetate, 2-phenyl ethanol, isoamyl alcohol, and other fruity volatiles, generating intensely fruity wheat beers when used in either sequential or mixed modes [64].

*Hanseniaspora guilliermondii* (Table 1), when applied first and followed by *S. cerevisiae*, can dramatically increase phenyl ethyl acetate levels (increased by 8.2 times), imparting pronounced rose and honey notes and improving the perceived body and balance of the beer [65]. Similar strategies with *H. vineae* and *M. pulcherrima* have been shown in related fermentations to boost fruity esters, β--damascenone, antioxidant capacity, and melatonin, suggesting that beers produced with these yeasts may offer functional benefits when consumed in moderation. During aging, co--fermentation with *B. bruxellensis* can generate complex mixtures of esters, phenols, acids, and terpene alcohols that contribute to the characteristic “Brett” profile, including spicy, barnyard, and fruity notes valued in lambic and gueuze styles [66]. *B. bruxellensis*, known for its characteristic “horse blanket” flavor and high acidity, plays a vital role in the mixed fermentation of Lambic and Gueuze beers [53]. *Brettanomyces* yeasts are particularly associated with the formation of 4-ethyl guaiacol and related phenolic compounds that add spicy and smoky nuances when controlled carefully [28]. Mixed fermentations including the probiotic yeast *S. cerevisiae* var. *boulardii* often end with this strain dominating cell populations; such beers can retain many viable probiotic cells, show increased antioxidant activity and polyphenol content, and maintain acceptable aroma comparable to beers produced with *S. cerevisiae* alone [67]. Postigo et al., [47] tested *H. vineae* and *M. pulcherrima* in mixed fermentation, and the beers were characterized by a greater body and balance, as well as fruity aromas and flavors. Moreover, the selected yeasts produced the highest levels of antioxidant capacity and melatonin during fermentation. All these features suggest that these beers can be considered functional for moderate consumption.

## 3. How Non-Conventional Yeasts Transform Beer

We can examine their metabolic contributions through three main chemical “engines”: the production of volatile phenols, the enzymatic release of hop-derived thiols, and the synthesis of esters and organic acids (Table 2).

The “wild” character of *Brettanomyces* is chemically defined by the transformation of hydroxycinnamic acids, which are naturally present in malt. While most brewing yeasts can decarboxylate these acids into vinyl-phenols (producing a simple clove-like aroma), *Brettanomyces* species possess a unique enzyme called vinyl phenol reductase (VPR).

In this pathway, ferulic acid is first converted to 4-vinylguaiacol. The VPR enzyme then uses NADH as a cofactor to reduce this intermediate into 4-ethylguaiacol (4-EG). Similarly, p-coumaric acid is reduced to 4-ethylphenol (4-EP). This specific reduction is a survival strategy for the yeast to maintain its internal redox balance NAD^++^/NADH^+^), but for the brewer, it results in the complex, “funky” aromas of leather, barnyard, and aged wood [68]. The species *L. thermotolerans* has gained popularity for its ability to naturally sour beer during primary fermentation. Unlike *Saccharomyces*, which convert most of the pyruvate into ethanol via acetaldehyde, *L. thermotolerans* utilizes the enzyme L-lactate dehydrogenase (LDH).

Through this pathway, pyruvate is diverted from the alcoholic fermentation route and reduced directly into L-lactic acid. This process consumes NADH and releases NAD^+^ allowing glycolysis to continue. The resulting beer has a lower pH and a clean, sharp acidity that avoids the “vinegar” notes associated with acetic acid, as *Lachancea* typically produces very low levels of volatile acidity compared to other wild yeasts. Many “tropical” aromas in modern beer are not originally present in the hops as free molecules but are bound to amino acids like cysteine or peptides like glutathione [69].

Non-conventional yeasts, particularly from the genus *Pichia* (such as *P. kluyveri*), are highly efficient at “unlocking” these compounds.

This is achieved through beta-lyase activity. The enzyme targets the carbon-sulfur bond of the odorless precursor (e.g., Cys-3MH), cleaving the molecule to release the free volatile thiol 3-mercaptohexan-1-ol (3MH). Chemically, this transformation is crucial because these thiols have incredibly low detection thresholds, meaning even trace amounts produced by the yeast can drastically change the sensory profile toward passion fruit and grapefruit.

Yeasts like *T. delbrueckii* and *H. uvarum* are often described as “ester powerhouses.” Their metabolism focuses heavily on the activity of Alcohol Acetyltransferases (AAT).

The chemical reaction involves the condensation of an acyl-CoA (usually Acetyl-CoA derived from sugar metabolism or fatty acid synthesis) with a higher alcohol (produced via the Ehrlich pathway from amino acids). *Hanseniaspora* species are particularly specialized in the production of 2-phenyl ethyl acetate, which is formed from the precursor 2-phenyl ethanol. This result in intense rose and floral aromas that are much more pronounced than those found in standard ales. Furthermore, *Torulaspora* yeasts tend to produce higher concentrations of ethyl esters (like ethyl hexanoate) which provide a “refined” fruity character resembling red apples or pineapple [70].

## 4. Beer Revolution with Non-Conventional Yeasts: The Healthy Issue

Functional beers were created to improve the health benefits of beverages by incorporating advantageous functional components and utilizing functional yeasts. Non-conventional yeasts can be extensively utilized to generate or convert advantageous compounds [71]. Among specialty beers, the most fascinating include low-calorie beer, low-alcohol or alcohol-free beer, beers with unique flavors, gluten-free beer, and functional beer. These unique beers are becoming more popular among consumers who are more focused on their health. The most notable non-conventional yeasts are the genera *Hanseniaspora*, *Pichia*, *Torulaspora*, and *Wickerhamomyces* (Table 1), among others, due to their varied enzymatic activities and their capacity to bio convert the components of the fermentation substrate. Craft beer is usually unfiltered and unpasteurized, making it a possible source of health advantages due to its beneficial compounds (antioxidants and polyphenols) and microorganisms [72,73] (Table 3).

### 4.1. Probiotic Beer as Vehicle for Probiotic

A novel functional beer is represented by probiotic beer, obtained by incorporating probiotic microorganisms. This strategy exploits evidence that craft beer is typically an unpasteurized and unfiltered matrix, thus representing a potential vehicle for delivering probiotics. Probiotics are defined as live microorganisms added to food, which, at certain doses, can be potentially beneficial for human health, especially in maintaining intestinal microbial balance [74]. Consequently, a probiotic beer is obtained by using probiotic microorganisms during the fermentation process. The best-known microorganisms utilized for their probiotic characteristics are lactic acid bacteria (LAB). Probiotics are not only bacteria, indeed, *S. cerevisiae* var. *boulardii* is a probiotic yeast strain that has been explored for specialty unfiltered and unpasteurized probiotic beer [57,67,75]. Studies have demonstrated that foods and drinks containing live probiotics are more effective in providing health benefits than products with inactive probiotics. Craft beer containing live yeasts can therefore be considered a new tool for beneficial health effects [76]. The use of several non-conventional genera such as *Brettanomyces*, *Debaromyces*, *Dekkera*, *Hansenula*, *Hanseniaspora*, *Kloeckera*, *Schizosaccharomyces*, *Sporobolomyces*, *Trichosporon*, *Torulopsis*, *Zygosaccharomyces*, *Lachancea*, *Kluyveromyces*, *Torulaspora*, *Metschnikowia*, *Kazachstania*, *Brettanomyces*, *Pichia*, *Candida*, *Hanseniaspora*, *Rhodotorula* and *Rhodosporidiobolus* was found to have great potential for use in the production of craft beers with probiotic characteristics [21,77]. Among these genera, several yeast strains have been investigated for their potential probiotic properties. Three non-conventional yeast strains belonging to the species *P. kluyveri*, *H. uvarum*, and *Candida intermedia* have demonstrated probiotic properties [78]. Since craft beer is generally unfiltered or unpasteurized, the yeast cells remaining after fermentation can impart a probiotic character to the beer and be considered beneficial to health. For this reason, craft rather than industrial brewing would be more appropriate, as viability is crucial for the efficacy of probiotics [67]. However, it should be noted that the presence of yeasts in beer, while contributing to a unique taste, can reduce its shelf life, as cell lysis during storage can negatively affect quality. Therefore, strain selection is fundamental in the production of craft beer.

One of the crucial points in probiotic beer production is the choice of when and how to introduce the chosen microbial strain or group. In fact, the addition can occur at different productions. The addition must clearly occur after any heat treatment to reach the required high cell concentration and ensure the viability of the live cultures [79]. Since a substantial concentration of live probiotic microorganisms is essential to the definition of a functional beverage, this requirement often gives craft breweries an advantage over industrial breweries [80]. Another strategy to obtain beer with functional characteristics could be the combination of functional or probiotic yeasts with functional ingredients [81]. Among these, promising ingredients include legumes such as chickpeas, lentils, and soy, which are important sources of protein for human health [22].

### 4.2. Healthy Yeast Metabolites

Beer contains a variety of beneficial substances, including antioxidants, protein (0.2–6.6 g/L), flavonoids (0.03–18.30 mg/L), polyphenols (34–426 mg/L), as well as small amounts of B group vitamins, macro and micronutrients, and soluble fiber [82]. These metabolites originate from the ingredients utilized in the beer-making process as well as from the microorganisms participating in fermentation. Certainly, throughout the fermentation process, yeasts generate metabolites that possess significant nutritional value and health-enhancing qualities. Wild yeast varieties can generate premium craft beers that are rich in nutrients, offer health benefits, and feature distinct flavor characteristics [21].

The use of non-conventional yeasts in beer production not only enhances organoleptic traits but also leads to beers that offer notable health benefits, featuring reduced alcohol and energy content along with increased levels of ascorbic acid, phenolic compounds, and antioxidant properties [83]. B vitamins are an important class of vitamins, essential for optimal physiological and neurological functions of human metabolism [84]. It has been widely demonstrated that yeasts may be able to synthesize vitamins B3, B6, and B9 [85,86,87]. Generally, beer brewed with traditional yeasts have sufficient B vitamin content to meet daily requirements; moderate beer consumption has even been found to have a specific positive impact on health because it can increase B9 and vitamin B12 intake [88,89]. Non-alcoholic beers fermented with a strain of *S. ludwigii*, two different strains of *Cyberlindnera saturnus* and *Kluyveromyces marxianus* have also been shown to contain B vitamins and can therefore be used as supplements to a balanced diet [84]. This type of beer can help people protect their health.

Low to moderate beer intake has demonstrated beneficial health impacts, promoting the growth of healthy microbiota. Multiple studies indicate that beer promotes gut microbiota by enhancing the growth of saccharolytic microorganisms that produce short-chain fatty acids [90].

### 4.3. Functional Yeasts

At present, numerous investigations have been carried out to assess the effectiveness of different non-traditional probiotic starter cultures in the production of craft beer [67,69,75]. *S. cerevisiae* var*. boulardii*, utilized in craft beer brewing, resulted in beers with decreased alcohol levels, enhanced antioxidant properties, comparable sensory characteristics to the commercial *S. cerevisiae* starter, significantly higher yeast viability after 45 days, and heightened acidification, which lowers contamination risks in large-scale brewing. Moreover, the probiotic yeast exhibited quicker growth and greater cell sizes compared to the commercial yeast, enhancing the probiotic mass in the final craft beer [75]. *S. cerevisiae* var*. boulardii* was likewise evaluated in combination with various *S. cerevisiae* strains during wort fermentation to create craft beers offering enhanced health advantages [69]. The application of these mixed fermentations did not harm beer aroma. It enhanced antioxidant activity and polyphenol levels compared to beers made with a single starter yeast, demonstrating the beneficial effect of the probiotic yeast strain on these aspects [67]. Furthermore, *S. cerevisiae* var*. boulardii* was used in mixed fermentation with lager starter strains, increasing its viability during 28 days of storage. Research on probiotic yeasts in craft beer has focused on non-conventional yeasts. Forty-three yeast strains belonging to the *genera Rodosporidobolus*, *Candida*, *Lachancea*, *Rhodotorula*, *Torulaspora*, *Kazachstania*, *Brettanomyces*, *Pichia*, *Kluyveromyces*, *Metschnikowia*, *Hanseniaspora* and *Saccharomyces*, previously validated for their probiotic characteristics, were tested for craft beer production [21]. In this research, hydrolyzed chickpea wort (PCW) or lentil wort (PLW) (substituting 20% of the wort) was incorporated into the wort utilized for craft beer production to enhance the protein levels in the finished beers. The chosen strains, *Kazachstania unispora*, *L. thermotolerans*, and *S. cerevisiae*, may serve as promising microbial options for creating an exceptional craft beer featuring elevated nutritional and functional traits along with a unique aroma [22]. The non-conventional yeasts used in beer brewing, along with their probiotic characteristics and effects on flavor, were assessed for the creation of specialty and healthier beers, including non-alcoholic/low-alcohol beers (NABLAB), low-calorie beers, and functional beers. These applications probably take advantage of the metabolic variations present in different non-traditional yeast species. Nonetheless, additional research is required to comprehend the mechanisms associated with the functional properties of non-conventional yeasts.

### 4.4. NABLAB

There is an increasingly massive customer demand for low-alcohol (0.5–1.2% *v*/*v*) and non-alcoholic (<0.5% *v*/*v*) beers (NABLAB). Various technological approaches can be used to produce these beers, such as physically removing alcohol from the beer or stopping fermentation with conventional brewing yeast, but in both cases, they lead to products with less-than-pleasant flavor profiles. Another strategy is the use of non-conventional yeasts, which exhibit a low or limited ability to ferment maltose, allowing them to produce low-alcohol beers while still maintaining aromatic complexity. *S. ludwigii* can produce non-alcoholic beers with rich aroma profiles thanks to its production of aromas (mainly esters and higher alcohols) [91]. *P. kluyveri* has a limited ability to ferment glucose, while significantly converting hop compounds into positive aroma compounds. This species produces medium levels of esters and high amounts of higher alcohols. Moreover, the co-fermentation of *S. cerevisiae* and *P. kluyveri* in a 1:10 ratio produced an ABV of 2.98% (*v*/*v*), exhibiting high concentrations of isoamyl acetate and phenyl ethyl acetate exhibiting banana, rose aromas. On the other hand, further studies are needed to clarify the relationship among some fermentation parameters, reducing sugar, and sensory properties [92]. Regarding *Z. rouxii*, the literature reports conflicting data regarding its application to obtain NABLAB beers [30,93] since the final content of ethanol found in the final product has been highly variable in the tests performed. De Francesco et al. [94] reported that beer fermented with the psychrophilic basidiomycete yeast *Mrakia gelida*, showed a low alcohol content of 1.40% (*v*/*v*) and a low diacetyl concentration (5.04 μg/L) contain more fruity aromas, such as apricot, grape, and lychee [94]. Low-alcohol beer produced by *M. gelida* is clear and yellow, with a fine and persistent head, full of intense and rich fruity aromas [94]. Methner et al. [84] used *Saccharomycopsis fibuligera,* to produce low-alcohol beer with an initial sugar content of 10 °P. In this way, a production of low-alcohol beer, approximately 0.8% (*v*/*v*) was obtained [84]. *Starmerella bombicola*, an important industrial producer of biosurfactants, was introduced for the first time into beer production [91]. Surprisingly, even at a temperature of 20 °C, this species was able to produce alcohol-free beer after 10 days of fermentation. This beer exhibited neutral aroma characteristics without any negative impact on sensory properties [75]. *Cyberlindnera saturnus*, a maltose-negative species, is suitable to produce non-alcoholic beer [31,95]. This yeast strain synthesizes a significant amount of isoamyl acetate, giving it a fruity aroma, predominantly reminiscent of banana and a unique flavor of red berries and apples, determined by its secondary metabolite β-damascenone [83,95]. For these reasons, the use of biological methods, based on the use of non-conventional yeasts as starter cultures, could be a valid tool for producing NABLAB. Indeed, these yeasts generally exhibit low capacity to ferment maltose (and maltotriose), the most abundant reducing sugar, in wort combining, at the same time, aroma enhancement and complexity improvement [17,75,95].

## 5. Potential Limitations and Future Perspectives

The use of non-conventional yeasts in craft beer as a tool for innovation has attracted great interest from both researchers and brewers. The safety assessment of non-conventional yeasts is a relevant aspect that needs to be investigated since a few yeast species are generally recognized as safe (GRAS/QPS) for use in food production [96]. In this regard, accurate taxonomic species identification and safety assessment should be evaluated. The production of high levels of histidine, phenylalanine, or tyrosine could increase the synthesis of biogenic amines with a risk to the health of the consumer [97]. Another relevant feature that needs to be investigated and clarified is the modality and feasibility of their use in breweries, taking in account the technological process and the possible interactions with the raw materials [97,98]. Considering these aspects, a guideline for the use of non-conventional yeasts in brewing is desirable. On the other hand, some non-conventional strains are already commercialized as starters for enology or brewing. They could release some amino acids, providing yeast assimilable nitrogen to *S. cerevisiae* during mixed fermentation.

## 6. Conclusions

**Figure 2:**
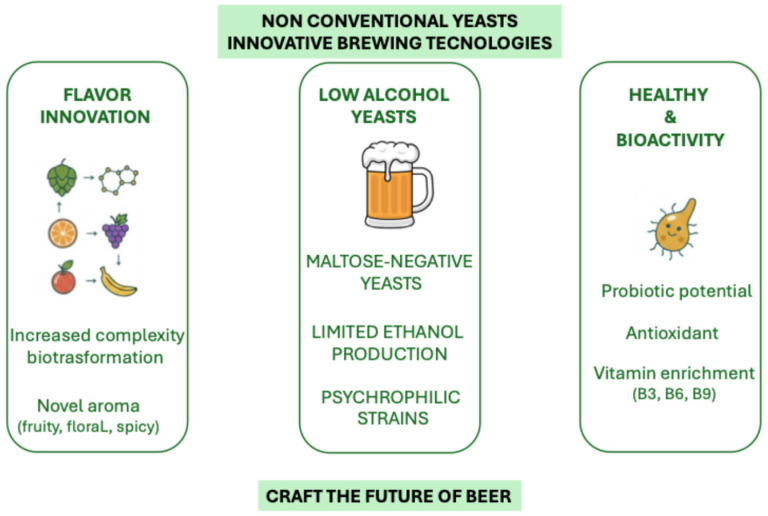
Innovative brewing technologies with non-conventional yeasts.

The use of non-conventional yeasts in craft beer production represents significant growth in the brewing sector, and beer could be a promising vehicle for innovation (Figure 2).

In contrast to traditional brewing, which typically relies on highly standardized strains of *S. cerevisiae* or *S. pastorianus* to ensure consistency and rapid turnover,—non-conventional yeasts not only improve the sensory profile of beer but also expand the potential for application and development in the brewing industry. Exploring non-conventional yeasts will play a fundamental role in shaping the future of beer. While traditional methods often prioritize predictable attenuation and technical efficiency, further investigations into their fermentation mechanisms, genomic and metabolic pathways, and their aroma contributions will provide valuable insights for brewers looking to innovate and differentiate their products in an increasingly competitive market. Furthermore, developing fermentation strategies that exploit the unique characteristics of these yeasts will further enrich the craft beer sector. The management of non-conventional yeasts provides the beer with further natural aromatic variations, such as complex esters and phenols—that differentiate it from mass-produced commercial beers, which often prioritize a neutral or uniform profile. Indeed, their use in pure culture, sequential culture, or with aeration during fermentation makes them very versatile. Furthermore, beer is composed of several bioactive compounds that not only give it sensory properties but also improve its functionality, in addition to providing health benefits, obviously when consumed responsibly. Functional beers will see the emergence of a health-conscious consumer who is attentive to the flavor that distinguishes this fermented beverage. Further research is needed to understand and develop the use of these yeasts in the brewing process, their probiotic and functional properties, and, above all, product stabilization strategies, which remain more challenging compared to the straightforward stabilization of traditional lagers and ales.

## Figures and Tables

**Table 1:** Fermentation characteristics of different non-conventional yeasts in brewing.

Yeast Species	Fermentation Characteristics	Brewing Application	References
*Torulaspora delbrueckii*	Ability to consume maltose the main sugar in wort and maltotriose. (strain-dependent) iso-alpha-acids have an inhibitory effect on some strains Volatile profile characterized by beta-phenyl ethanol (“rose” flavors), n-propanol, iso-butanol, amyl alcohol (“solvent brandy” aroma), and ethyl acetate. Bio-transforms monoterpenoid flavour compounds of hops (e.g., reduction in geraniol to citronellol).	Suitable for low-alcoholic beer production due to its aromatic Fruit/citric” and “fruity/ester” notes and “full-bodied” attributes in pure or mixed fermentations with *S. cerevisiae*	[14,15,16,17,18]
*Lachancea thermotolerans*	Production of lactic acid. Excellent attenuation and sensory characteristics. Exhibited good competition in co-cultures with *S. cerevisiae*. A general reduction in acetaldehyde content in all mixed fermentations. Enhancement of ethyl butyrate and ethyl acetate. Showed interesting probiotic features and is presumed of safety (QPS). Proposed to recycle brewer’s spent grains (BSG) for non-alcoholic and low-alcohol beer (NABLAB).	Production of sour beer. Viable alternative to lactic acid bacteria for sour beer production, avoiding the refermentation process. Contributes to the overall quality and dryness of the beer. Improves beer quality by reducing an undesirable off-flavor. Contributes to fruity and ester notes in the resulting beers. Proposed to enhance the functionality of craft beer. Offers a sustainable use for a brewing byproduct in low-alcohol production.	[19,20,21,22,23,24]
*Wickerhamomyces anomalus* (formerly *Pichia anomala*)	Resistant to unfavourable environmental conditions. Produces ethyl acetate, ethyl propanoate, phenyl ethanol, 2-phenyl ethyl acetate. Imparts fruity character or unpleasant solvent-like character	Used in sequential or co-inoculation with *S. cerevisiae* starter strains. Variable ability to ferment maltose.	[25,26,27]
*Pichia kluyveri*	Limited ability to ferment glucose. Contribute fruity banana flavors. Also produces compounds that give undesirable flavors (requiring sequential fermentation). Produces average levels of esters and high quantities of higher alcohols. Significantly changes hop compounds into positive flavor compounds.	Potential for low-alcohol or alcohol-free beers due to limited glucose fermentation. Commercial use not yet implemented.	[28]
*Pichia kudriavzevii*	Extremely efficient in the consumption of sugars and the generation of ethanol (in specific Belgian-style pale ale trial). Produces higher alcohols like 1-octanol and 1-pentanol (responsible for orange, rose, and bread aromas). Isoamyl alcohol (a higher alcohol) was reported to impart fruity flavors.	Used to produce light craft beer with low bitterness and neutral characteristics. Selected strain produced a medium level of ethanol (5.2% *v*/*v*) in a Belgian-style pale ale.	[14]
*Pichia* Genera (General)	-	Tested for reusing brewer’s spent grains (BSG) to obtain another beer with low or no alcohol, showing promising results.	[13]
*H. valbyensis* & *H. vineae*	Evaluated for application in the production of non-alcoholic beer (NABLAB).	Major limitation for use as a primary ale yeast, as maltose is the main sugar in wort.	[19,29]
*Hanseniasporauvarum* (Co-starter)	Selected strains might be applied as co-starters for producing beer up to 10% (*v*/*v*) ethanol.	*H. vineae* strain tested its ability to acidify the wort in the production of sour beer.	[30]
*Zygotorulaspora florentina*	Increased higher alcohols, isoamyl acetate, and alpha-terpineol content.	Evaluated in pure and mixed fermentation with *S. cerevisiae*. Contributes to the aroma.	[30]
*Saccharomycodes ludwigii*	Unable to catabolize maltose. Low ethanol production (0.51% to 1.36% *v*/*v*).	Suitable for low-alcohol beer production.	[31]
*Zygosaccharomyces rouxii*	Low ethanol production (0.93% *v*/*v*). Exhibited resistance to ethanol and hop.	Produced positive aromatic characteristics. Relevant features for brewing processes, including NABLAB.	[31]
*Cyberlindnera* genus	-	Screened to produce fruity non-alcoholic beer (NABLAB).	[14]
*Pichia kudriavzevii* & *Meyerozyma guilliermondii*	Suitable to produce volatile compounds like ethyl acetate, 2-phenyl ethanol, and isoamyl alcohol.	Contributes fruity notes and floral nuances to mixed fermentations (isolated from Belgian wheat beer sludge).	[32]
*Zygoascus meyerae*	Produced 4-vinylguaiacol, beta-phenyl ethyl alcohol, and isoamyl alcohol at levels significantly above the sensory threshold.	Interesting for wheat and blond craft beer styles.	[14]
*Candida zemplinina* (now *Starmerella bacillaris*)	Found to be a promising starter (in mixed fermentation with *S. cerevisiae*) to produce low-alcohol beers.	Yielded pleasant organoleptic characteristics in all media tested (Pilsner, Weizen, Amber).	[33]
*Starmerella bombicola*	Important industrial producer of biosurfactants. Able to produce non-alcoholic beer	Resulted in neutral and no negative impact on organoleptic properties of NABLAB beer.	[14]
*Lindnera jadinii* & *L. saturnus*	-	Produced banana-flavored beers with low alcohol content. Promising strains for NABLAB.	[33]
*Kazachstania servazzii, Kluyveromyces marxianus, Pichia fermentans*	Tested (along with *T. delbrueckii* isolates from sourdough) for cold contact fermentations to reduce wort aldehydes	Only *T. delbrueckii* showed promising results in this specific low-temperature context.	[14]
*Candida pulcherrima*	-	Investigated for potential in non-alcoholic beer production.	[14]

**Table 2:** Biochemical mechanisms of the main non-conventional yeasts involved in brewing process innovation.

Yeast	Key Enzyme(s)	Chemical Pathway/Precursor	Resulting Compounds	Sensory Profile
*Brettanomyces* spp.	Vinyl Phenol Reductase (VPR)	Reduction of Hydroxycinnamic acids (Ferulic & p-Coumaric acid)	4-ethylguaiacol (4-EG) & 4-ethylphenol (4-EP)	“Funky,” leather, barnyard, aged wood
*Lachancea thermotolerans*	L-lactate dehydrogenase (LDH)	Diversion of Pyruvate from the alcoholic route	L-lactic acid	Clean, sharp acidity; lower pH without vinegar notes
*Pichia* (e.g., *P. kluyveri*)	β-lyase	Cleavage of C-S bonds in bound precursors (e.g., Cys-3MH)	Free volatile thiols (e.g., 3-mercaptohexan-1-ol)	Tropical fruit, passion fruit, grapefruit
*Hanseniaspora uvarum*	Alcohol Acetyltransferases (AAT)	Condensation of Acetyl-CoA + 2-phenylethanol	2-phenyl ethyl acetate	Intense rose and floral aromas
*Torulaspora* *delbrueckii*	Alcohol Acetyltransferases (AAT)	Condensation of Acyl-CoA + higher alcohols	Ethyl esters (e.g., ethyl hexanoate)	Refined fruity notes, red apple, pineapple

**Table 3:** Non-conventional yeasts with health properties.

Functional Traits	Yeast Strains/Genera	Health Benefits
Probiotic Yeasts	*S. cerevisiae* var. *boulardii*, *P. kluyveri*, *H. uvarum*, *Candida intermedia*	Maintains intestinal microbial balance; high antioxidant activity; increased polyphenol levels; high cell viability during storage.
Low-Alcohol/NABLAB	*Saccharomycodes ludwigii*, *Pichia kluyveri*, *Mrakia gelida*, *Saccharomycopsis fibuligera*, *Starmerella bombicola*, *Cyberlindnera saturnus*	Produces non-alcoholic (<0.5%) or low-alcohol (0.5–1.2%) beer; contributes B vitamins (B3, B6, B9).
Healthy metabolites	*Hanseniaspora*, *Torulaspora*, *Wickerhamomyces*, *Lachancea*, *Kluyveromyces*, *Brettanomyces* *Kazachstania unispora*, *L. thermotolerans*, *S. cerevisiae* (mixed)	Bio-converts fermentation substrates into advantageous compounds; higher levels of ascorbic acid and antioxidants. Elevated nutritional value when combined with functional ingredients like legumes (chickpeas, lentils).

## Data Availability

No new data were created or analyzed in this study. Data sharing is not applicable to this article.
